# The P134Q Sucrose Phosphorylase: Subtle Changes in the Catalytic Properties Benefit the Production of 2‐*O*‐α‐Glucosyl Glycerol

**DOI:** 10.1002/bit.70003

**Published:** 2025-06-20

**Authors:** Alexander Sigg, Mario Klimacek, Martin Pfeiffer, Jorick Franceus, Tom Desmet, Bernd Nidetzky

**Affiliations:** ^1^ Institute of Biotechnology and Biochemical Engineering Graz University of Technology, NAWI Graz Graz Austria; ^2^ Austrian Centre of Industrial Biotechnology (acib) Graz Austria; ^3^ Centre for Synthetic Biology (CSB), Unit for Biocatalysis and Enzyme Engineering, Faculty of Bioscience Engineering Ghent University Ghent Belgium

**Keywords:** enzyme engineering, model‐based optimization, regiocontrol, sucrose phosphorylase, transfer reaction, transglycosidase, window‐of‐operation analysis

## Abstract

Protein engineering of *Bifidobacterium adolescentis* sucrose phosphorylase (*Ba*SucP) has previously identified the P134Q enzyme variant for site‐selective glycosylation at the 2‐OH of glycerol. Besides improvement in selectivity, the P134Q‐*Ba*SucP additionally involves enhanced affinity for glycerol as a biochemical property potentially important for the production of 2‐*O*‐α‐glucosyl glycerol (2GG), a commercialized skincare ingredient for cosmetic applications. Here, we performed a detailed kinetic model‐based evaluation of P134Q‐*Ba*SucP in initial‐rate and full reaction time course analyses to obtain a mechanistic interpretation and a comprehensive assessment of the process improvements achievable by the P134Q variant compared to the native enzyme. We show that P134Q‐*Ba*SucP involves ∼50‐fold lowered reactivity with phosphate compared to native enzyme. The effect likely arises from decreased conformational flexibility of the substrate binding pocket in the P134Q variant that may also serve to constrain the positioning of glycerol for glycosylation. Glycerol reactivity is decreased ∼1.3‐fold in P134Q‐*Ba*SucP; yet because the hydrolytic reactivity is lowered even more (threefold), the transfer efficiency to glycerol of the variant is enhanced ∼twofold compared to the native enzyme. Product inhibition by 2GG is decreased ∼threefold in P134Q‐*Ba*SucP. These properties of P134Q‐*Ba*SucP combine into major benefits for 2GG synthesis in terms of productivity and product yield. Model‐based window‐of‐operation analysis for 2GG production from sucrose and glycerol further reveals the significant potential for saving on the excess glycerol used in the process that results from replacing the wild‐type *Ba*SucP with the P134Q variant. Collectively, this study shows the important interplay of enzyme and reaction engineering in the optimization of glycoside production through biocatalytic transglycosylation.

## Graphical abstract

Production of the cosmetic ingredient 2‐α‐d‐glucosyl glycerol (2GG) by the Pro134Gln variant of sucrose phosphorylase from *Bifidobacterium adolescentis* is reported. The authors show with detailed characterization and hybrid kinetic modeling that the enzyme variant can improve the 2GG productivity of the biocatalytic process due to its increased selectivity and enhanced transfer/hydrolysis reactivity. The 2GG production by transglycosylation of glycerol from sucrose requires lower amounts of glycerol which will facilitate the downstream processing of 2GG.

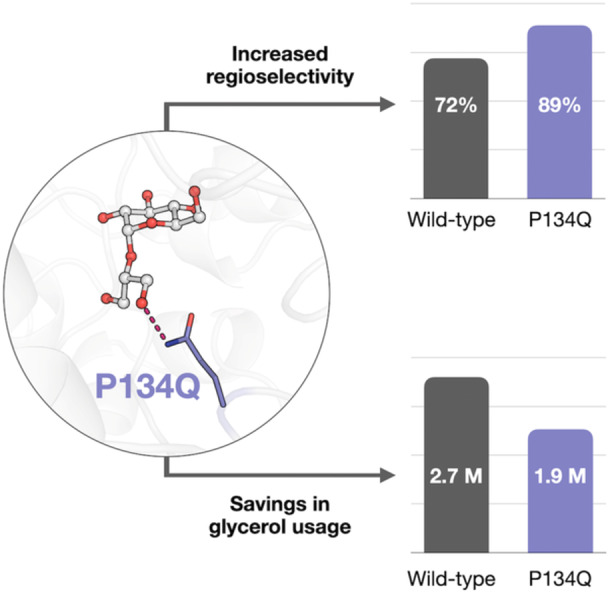

**Abbreviations**
2GG: 2‐*O*‐α‐d‐glucosyl glycerol
*Ba*SucP: SucP from *Bifidobacterium adolescentis*
G1P: α‐d‐glucose 1‐phosphate
GG: α‐d‐glucosyl glycerol
Glc: glucose
GOH: glycerol
Pi: phosphate
Suc: sucrose
SucP: sucrose phosphorylase
WT: wild‐type enzyme

## 1 Introduction

Glycosylation is an important synthetic transformation in the industrial production of glycosides and oligosaccharides (de Roode et al. 2003; Seibel and Buchholz 2010). Enzymes are powerful catalysts that promote glycosylation with high efficiency and selectivity (Desmet et al. 2012; Li et al. 2022; Nidetzky et al. 2018). Among the enzyme classes known for glycosylation (glycosyltransferases EC 2.4; glycoside hydrolases EC 3.2), the so‐called transglycosidases have received special interest for industrial application on the basis of the robust process technologies that these enzymes make possible (Franceus and Desmet 2020; Mészáros et al. 2021; Moulis et al. 2021). Transglycosidases are glycoside hydrolases that catalyze the transformation of one glycoside into another. Their reactions use a glycosidic substrate (termed “donor”) to glycosylate a second substrate (termed “acceptor”). Being glycoside hydrolases by evolutionary origin and mechanism used, transglycosidases catalyze the hydrolysis of glycosides as well (Mészáros et al. 2021; Moulis et al. 2021). Hence, suppressing hydrolysis of the donor substrate and/or the glycosidic product is a very important goal in this field (Adlercreutz 2017; Bissaro et al. 2015; Danby and Withers 2016; de Roode et al. 2003). A wealth of protein engineering studies has previously aimed at creating hydrolysis‐deficient transglycosidases for improved glycoside synthesis (Bissaro et al. 2015; David et al. 2019; Lundemo et al. 2017; Teze et al. 2014; Teze et al. 2021). Similarly in reaction engineering, many studies describe process intensification by targeting conditions (“process windows”) of low hydrolysis within the overall parameter space of the transformation used (Chen et al. 2020; Fagundes et al. 2022; Guerrero et al. 2015; Hoffmann et al. 2020; Klimacek et al. 2020; Sigg et al. 2021; Vera et al. 2016). Integrated approaches that combine protein and reaction engineering for process optimization are less well explored though (Ruzic et al. 2020; Schmölzer et al. 2019; Zeuner et al. 2014; Zeuner et al. 2019). The productive interplay of molecular and process‐related factors of conversion efficiency is, however, critical for the superior performance of an enzymatic glycosylation. Understanding the important interconnection of enzyme characteristics and reaction parameters in contributing to the overall process efficiency is central at all stages of the development (Klimacek et al. 2020). It provides the essential basis for process decision making, including selection of the best‐suited biocatalyst from an available set of transglycosidase enzymes. Here, we demonstrate such a kind of integrated engineering approach for the enhanced production of 2‐*O*‐α‐d‐glucosyl glycerol (2GG) via direct glycosylation of glycerol from sucrose (Goedl et al. 2008; Klimacek et al. 2020; Sigg et al. 2021). 2GG is a natural osmolyte that is produced industrially by a transglycosidase process analogous to the one investigated here (Kruschitz and Nidetzky 2022; Luley‐Goedl et al. 2010). 2GG has been commercialized as skincare ingredient for cosmetic applications. The enzyme used to synthesize 2GG is sucrose phosphorylase (SucP; EC 2.4.1.7), a transglycosidase of CAZy family GH13 (Franceus and Desmet 2020). Recently, an alternative biocatalytic route to 2GG was proposed that proceeds via intermediary α‐d‐glucose 1‐phosphate (G1P), generated by SucP and used by a specific glucosylglycerol phosphorylase (Franceus et al. 2018; Zhang et al. 2022; Zhang et al. 2020). That one‐pot two‐enzyme route is not discussed here.

**Figure 1:**
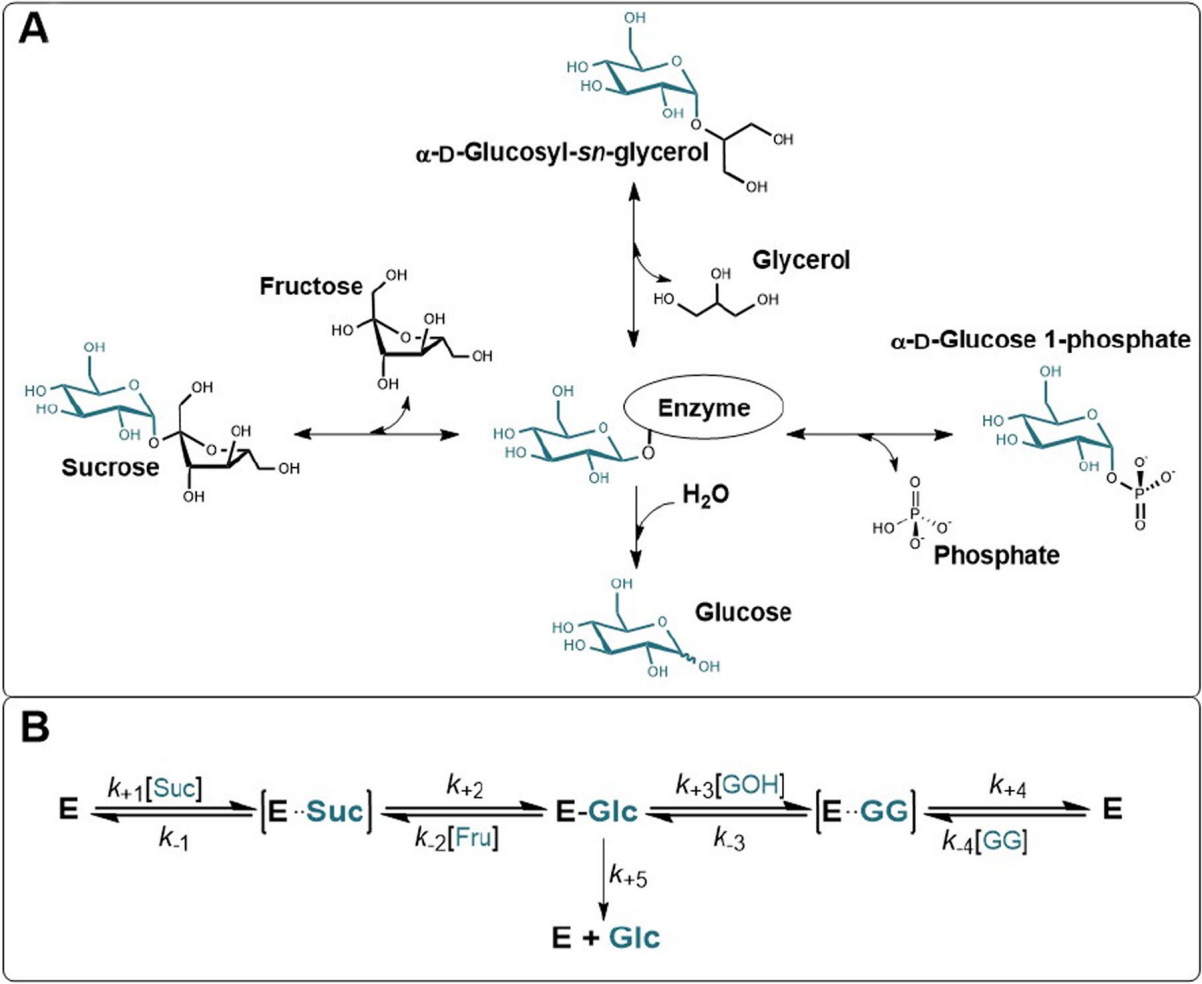
Reactions of SucP analyzed in this study. (A) Chemical reaction scheme and (B) proposed kinetic mechanism of transglycosylation and hydrolysis (Sigg et al. 2021). Panel A shows 2GG but depending on the enzyme used, 1GG can also be produced.

Figure 1A shows the main reactions catalyzed by SucP with sucrose (Suc) as donor (Vyas and Nidetzky 2023). These all start with the formation of a covalent β‐glucosyl enzyme intermediate as a common first step, but diverge at the second step. In the native reaction, that is often termed phosphorolysis, the intermediate reacts with phosphate to give G1P. The overall phosphorolysis is a freely reversible process. In the transglycosylation to yield 2GG, the reaction is performed with glycerol (GOH) as acceptor and no phosphate is present. The 2GG formation is only weakly reversible compared to the G1P formation (Sigg et al. 2021). During hydrolysis, the intermediate reacts with water, which is the sole reaction in the absence of acceptor. It can be reduced to a mere side reaction when the acceptor is present at a suitable concentration. Glycerol is much (≥ 10^3^‐fold) less efficient than phosphate in suppressing hydrolysis and has to be present in very high concentrations (≥ 1 mol/L), typically in excess over the sucrose used during 2GG production (Goedl et al. 2008; Klimacek et al. 2020; Sigg et al. 2021). We return to this point later in the paper when discussing process windows for 2GG production.

The SucP enzymes that were well‐characterized for 2GG synthesis divide according to characteristics of glycerol regioselectivity and temperature stability. The enzyme from *Leuconostoc mesenteroides* (*Lm*SucP) produces almost exclusively the desired 2GG (≥ 90%), whereas the enzyme from *Bifidobacterium adolescentis* (*Ba*SucP) produces a ∼2:1 mixture of 2‐ and 1‐isomer (1GG) of *O*‐α‐d‐glucosyl glycerol (Franceus et al. 2021; Schwaiger et al. 2021). The regio‐isomeric mixture of total product is hereinafter referred to as GG. Concerning temperature stability, *Ba*SucP is much more robust than *Lm*SucP. In an effort to combine both desirable traits into a single enzyme, *Ba*SucP was subjected to extensive engineering (Franceus et al. 2021). The P134Q variant was identified for its superior glycerol regioselectivity (∼88% 2GG) close to that of *Lm*SucP. Importantly, wild‐type thermostability was retained in P134Q‐*Ba*SucP. The P134Q variant also showed enhanced kinetic properties for glycerol (*K*_m_ lowered 5.5‐fold; *V*_max_/*K*_m_ increased 21‐fold). Overall, therefore, P134Q‐*Ba*SucP appeared as a promising candidate for 2GG production (Franceus et al. 2021). We note that progress has also been made in engineering *Lm*SucP for thermostability (Ma et al. 2024; Yang et al. 2022). However, there still remains a huge gap in thermotolerance between the two enzymes. For example, the recently reported *Lm*SucP variants V23L and V23L/S424R show a half‐life of approximately 1 h at 50°C (Yang et al. 2022), whereas P134Q‐BaSucP retains all activity for at least 3 days at that temperature. Very recently, engineering of the 2‐OH regioselectivity in glycerol of the SucP from *Limosilactobacillus reuteri* was reported. Zhu et al. (2025) showed a double variant (R137M/L337T) of the enzyme with substantially improved selectivity (98.0%) compared to the native SucP (84.7%). The stability of the *Li. reuteri* SucP has, however, not been studied in detail to our knowledge.

The current study of P134Q‐*Ba*SucP builds on Sigg et al. (2021), who presented a detailed hybrid kinetic model for the glycosylation of glycerol from sucrose at industrially relevant substrate concentrations. The term hybrid was used to indicate the microscopic‐level extension of the mechanism‐based kinetic model for the enzymatic reaction (Figure 1B; Klimacek et al. 2020) by the empirical description of the general solute effect of substrates and products used at molar concentrations. The hybrid model was shown to present a powerful engineering tool for the rigorous assessment of *Lm*SucP and *Ba*SucP regarding GG production. In their study, Sigg et al. gave a cautionary notice on the use of simple kinetic parameters from initial‐rate characterization (e.g., *K*_m_) to predict enzyme conversion efficiency. Here, we therefore decided to evaluate P134Q‐*Ba*SucP in the extensive way as shown previously for the wild‐type enzyme. Results of a detailed kinetic model‐based characterization of P134Q‐*Ba*SucP in initial‐rate and full reaction time course analyses are presented. The findings provide enhanced mechanistic interpretation of the consequences of the site‐directed substitution in *Ba*SucP and allow for a comprehensive appraisal of the process improvements achievable by the P134Q variant. Overall, we show how the enhanced glycerol regioselectivity and other subtle changes in the kinetic properties of P134Q‐*Ba*SucP combine to a significant benefit for the production of 2GG analyzed in small‐scale reactions. The feasibility of scale‐up of the enzymatic reaction with transfer to an industrial process was demonstrated for the production of 2GG using *Lm*SucP as the biocatalyst (Kruschitz and Nidetzky 2022; Luley‐Goedl et al. 2010).

## 2 Materials and Methods

### 2.1 Materials Used

Chemicals were of the highest purity available from Sigma‐Aldrich (Vienna, Austria) or Carl Roth (Karlsruhe, Germany). Purified WT‐*Ba*SucP (GenBank identifier AF543301.1) and P134Q‐*Ba*SucP (Franceus et al. 2021) were prepared by methods reported in Klimacek et al. (2020) and references cited therein. Briefly, the enzymes were obtained from *Escherichia coli* overexpression cultures and were purified via their C‐terminal His‐tag. Standard enzyme activities (U, units) refer to an activity assay performed at 30°C in 50 mM MES buffer (pH 7.0). Suc (20 mM) and GOH (2.0 M) were used as substrates, and the release of fructose (Fru) was measured enzymatically in sample taken from the reaction at several times (5, 10, 15, 25, and 40 min; see Section 2.2 below). The rate was determined from the linear change of [Fru] with time. One U (unit) is the enzyme amount that releases 1 µmol Fru/min in the standard conditions. Purified preparations of WT‐*Ba*SucP and P134Q‐*Ba*SucP showed specific activities of 15.3 (±1.1; *N* = 2) and 11.4 (±0.2; *N* = 2) U/mg, respectively.

### 2.2 Enzymatic Reactions

Reactions were performed at 30°C in 50 mM MES buffer (pH 7.0) using Eppendorf tubes (1.5 mL liquid volume) incubated on an Eppendorf ThermoMixer C with agitation at 750 rpm. Samples (200 μL) were withdrawn at appropriate times and quenched in the same volume of preheated MES buffer (99°C). Samples were immediately transferred to a boiling water bath for an additional 10 min of heat inactivation. After centrifugation, samples were stored at −20°C until further analysis.

### 2.3 Initial Rates

The reactions analyzed were the following: phosphorolysis of Suc; transfer to GOH; and hydrolysis (see Figure 1). For transfer to GOH and hydrolysis, Suc and G1P were used as donor. The substrate concentrations used are indicated immediately with the results. Incubations were done as described in Section 2.2. Enzymatic reactions. Up to five samples were withdrawn in 40 min of reaction. WT‐*Ba*SucP and P134Q‐*Ba*SucP were applied at 2–8 µg/mL and 5–47 µg/mL, respectively. The enzyme concentration was varied to account for changes in activity depending on the reaction analyzed. Substrate conversion was limited to below 15%.

Product or products released in the respective reaction were determined by photometrical assays using a Beckman Coulter DU 800 spectrophotometer. The assays used were described concisely in Klimacek et al. (2020) and the Supporting Information associated with that paper. Phosphorolysis of Suc was monitored by G1P release (enzymatic assay; Vyas and Nidetzky 2023). Glucose release (enzymatic assay) was measured additionally to record accompanying hydrolysis. Transfer to GOH from Suc was monitored by the release of Fru and glucose (Glc), measured with an enzymatic assay. Transfer to GOH from G1P was monitored by release of phosphate (Pi; colorimetric chemical assay; Vyas and Nidetzky 2023) and Glc.

Volumetric initial rates [mM/min] were determined from linear time courses of product release. They were divided by the molar enzyme concentration used to obtain apparent turnover frequencies [1/s]. The molar enzyme concentration was calculated from the protein concentration with the enzyme molar mass of 56 kDa (Schwaiger et al. 2021). Protein was measured with the Bradford assay referenced against BSA.

### 2.4 Kinetic Parameters

Apparent kinetic parameters (maximum rates Vapp [s^−1^], Michaelis constants Kapp [mM]) were determined from nonlinear least‐squares fits of one of the Equations (1)–(4) to the appropriate set of initial‐rate data. The fitting was performed in OriginPro 2019b. Equations (1)–(4) are phenomenological mathematical relationships taken from Sigg et al. (2021). The initial rates v (all in [s^−1^]) are shown with subscript and superscript on the right (e.g., $v_{\text{Fru}}^{\left[\text{GOH}\right]}$) to indicate, respectively, the product measured (e.g., Fru) and the constant concentration of a second substrate used (e.g., [GOH] in the transfer reaction). $v_{H}$ is the hydrolysis rate based on measurement of Glc release. Superscript “app” to the left of V or K indicates the apparent nature of the kinetic parameter. Subscript in Vfapp refers to the forward (f) direction of reaction from Suc, and VGGapp refers to the reaction from GG as donor. Subscript in Kapp (e.g., KSucapp) indicates the varied substrate referred to. The transfer coefficient TC [M^−1^] refers to the dependence of the rate ratio $v_{\text{GG}}/v_{H}$ on [GOH]. Note the requirement of mass balance in the transfer reaction from Suc, namely that $v_{\text{Fru}}=v_{\text{GG}}+v_{H}$.

(1) vFru[GOH]=[Suc]·VappfKappSuc+[Suc],

(2) vFru[Suc]=Vfapp⋅[GOH]KGOHapp+[GOH]+vSucH[GOH]=0,

(3) $$\frac{v_{\text{Fru}}}{v_{H}}=\mathrm{TC}\cdot \left[\text{GOH}\right]+1,$$

(4) vG1P[Pi]=[GG]⋅VGGappKGGapp+[GG].

### 2.5 Reaction Time Courses

Experiments were performed at concentrations of Suc (≥ 300 mM) and [GOH] (≥ 1.0 M) relevant for industrial production of 2GG. Enzymes were applied at constant volumetric activity of 3.7 U/mL (standard assay). Enzymes were shown to be stable throughout the conversion experiments. The experiments were performed as described in Section 2.2. Enzymatic reactions. Samples were analyzed by HPLC using a Merck Hitachi L‐7100 system (Merck) equipped with refractive index detection and operated with a YMC‐Pack Polyamine II/S‐5 µm/12 nm column (YMC). All reactants (i.e., Suc, GOH, Fru, Glc, 1GG, 2GG) were quantified. The system was used in isocratic mode (acetonitrile:water, volume ratio 3:1) at ambient temperature and with a flow rate of 1 mL/min (Kruschitz and Nidetzky 2020).

### 2.6 Enzyme Structure Modeling

The crystal structure of WT‐*Ba*SucP (pdb: 2GDV) contains bound β‐d‐glucose (Mirza et al. 2006). Yasara v. 18.2.7 (Land and Humble 2018) was used to convert the β‐d‐glucose into G1P. The G1P pose was optimized by global energy minimization with AmberFB‐15 force field (Stoppelman et al. 2021). The results agreed well with the earlier model of Mirza et al. (2006).

### 2.7 Kinetic Model Fitting

The hybrid kinetic model used (Sigg et al. 2021) is shown in Supporting Information S1: Table S1. The model was fitted to combined data from initial rate and time course measurements of P134Q‐*Ba*SucP using the stepwise procedure of Sigg et al. (2021). Briefly, the first step involved fitting of initial rate data of the forward reaction, that is, the dependencies of $v_{\text{Fru}}$ on [Suc] and [GOH], $v_{H}$ on [Suc], and $v_{\text{Fru}}/v_{H}$ on [GOH], using the Microsoft Excel Solver add‐in. Detailed Solver settings are summarized in Sigg et al. (2021). Results from initial rate experiments together with kinetic parameters from fits of Equations (1)–(4) served as model constraints. Simulation of the dependency of $v_{H}$ on [GOH] was used as internal control.

The best‐fit results of the first step served as start values and model restrictions in the fit of the reaction time courses, as summarized in Supporting Information S1: Table S2. Fitting the reversible full kinetic model in Supporting Information S1: Table S1 was done with the parameter estimation tool embedded in COPASI 4.29 (Hoops et al. 2006). The program uses an evolutionary strategy with stochastic ranking (SERS) as an optimization method. The algorithm was set to a total of 6000 generations and a population size of 60. Other parameters of the Solver were kept at standard settings.

### 2.8 Simulation and Model‐Based Window of Operation Analysis

Extensive simulation of reaction time courses for GG synthesis from Suc and GOH was done with the parameter scan tool in COPASI. Initial concentrations of [Suc] and [GOH] were varied from 0.1 M and 0.0 M in increments of 100 mM up to 2.00 and 4.00 M, respectively. For further analysis, the reaction time and the enzyme loading were set constant at 48 h and 11.1 U/mL. The parameters analyzed were Suc conversion ($X_{\text{Suc}}$), GG yield ($Y_{\text{GG}}$), and GG concentration. The results defined a window of operation for further interpretation with respect to different process tasks.

## 3 Results and Discussion

### 3.1 Kinetic Characterization of P134Q‐*Ba*SucP

To obtain a preliminary picture of the kinetic consequences resulting from the substitution of Pro134 by Gln, we analyzed P134Q‐*Ba*SucP in standard assays for the reversible phosphorolysis of Suc, transfer to GOH, and hydrolysis (Figure 1A). To allow for direct comparison with our earlier studies of WT‐*Ba*SucP (Klimacek et al. 2020; Sigg et al. 2021), assays were performed at 30°C. Apparent turnover numbers ($v_{X}$, $v_{H}$) of P134Q‐*Ba*SucP in these reactions are summarized in Table 1 along with the corresponding values for the wild‐type enzyme. Results for WT‐*Ba*SucP are validated by close agreement with the literature (Aerts et al. 2011; Schwaiger et al. 2021). P134Q‐*Ba*SucP was impaired considerably (53‐fold) in the forward phosphorolysis of Suc. The reverse reaction from G1P (“synthesis” in Table 1) was impaired in a similar degree (66‐fold). Interestingly, the hydrolysis rate $v_{H}$ with Suc was hardly affected (1.67‐fold) by the site‐directed substitution (Table 1). The reaction selectivity $v_{X}$/$v_{H}$ was changed 32‐fold from ~83 in WT‐*Ba*SucP to only ~2.6 in P134Q‐*Ba*SucP.

**Table 1:** Comparison of WT‐*Ba*SucP and P134Q‐*Ba*SucP based on activities for different reactions catalyzed.

Reaction type	Donor	Acceptor	WT			P134Q		
			Transfer ($v_{X}$)	Hydrolysis ($v_{H}$)	$v_{X}/v_{H}$	Transfer ($v_{X}$)	Hydrolysis ($v_{H}$)	$v_{X}/v_{H}$
	[mM]	[mM]	[1/s]	[1/s]	[‐]	[1/s]	[1/s]	[‐]
Phosphorolysis	250^a^	50^b^	75.5 ± 2.2	0.9 ± 0.1	82.8 ± 0.1	1.43 ± 0.05	0.56 ± 0.02	2.55 ± 0.05
Synthesis	100^c^	100^d^	42.3 ± 1.9	n.d.	n.d.	0.64 ± 0.02	n.d.	n.d.
Transglucosylation Suc	20^a^	2000^e^	14.3 ± 1.0	0.8 ± 0.03	17.9 ± 0.1	10.6 ± 0.2	0.45 ± 0.03	23.6 ± 0.1
Transglucosylation G1P	50^c^	2000^e^	8.6 ± 0.2	0.48 ± 0.08	17.9 ± 0.2	0.58 ± 0.01	0.028 ± 0.011	20.7 ± 0.4

*Note:* n.d., not determined.

^a^ Suc.

^b^ Pi.

^c^ G1P.

^d^ Fru.

^e^ Glycerol analyzed products: G1P, Glc (phosphorolysis); Pi, Glc (synthesis); Fru, Glc (transglucosylation sucrose), Pi, Glc (transglucosylation G1P).

Transglycosylation of GOH was analyzed with Suc or G1P as donor. Using Suc, the transfer rate $v_{X}$ and the hydrolysis rate $v_{H}$ of P134Q‐*Ba*SucP were both slightly lower than the corresponding rates of WT‐*Ba*SucP. The rate ratio $v_{X}$/$v_{H}$ was similar for both enzymes, with only a slight benefit (1.32 = 23.6/17.9) noted for P134Q‐*Ba*SucP. Using G1P, both $v_{X}$ and $v_{H}$ were substantially ($v_{X}$: 15‐fold; $v_{H}$: 17‐fold) decreased in the variant enzyme. Interestingly, however, the $v_{X}$/$v_{H}$ ratio was similar for both enzymes and comparable between the two donor substrates.

**Figure 2:**
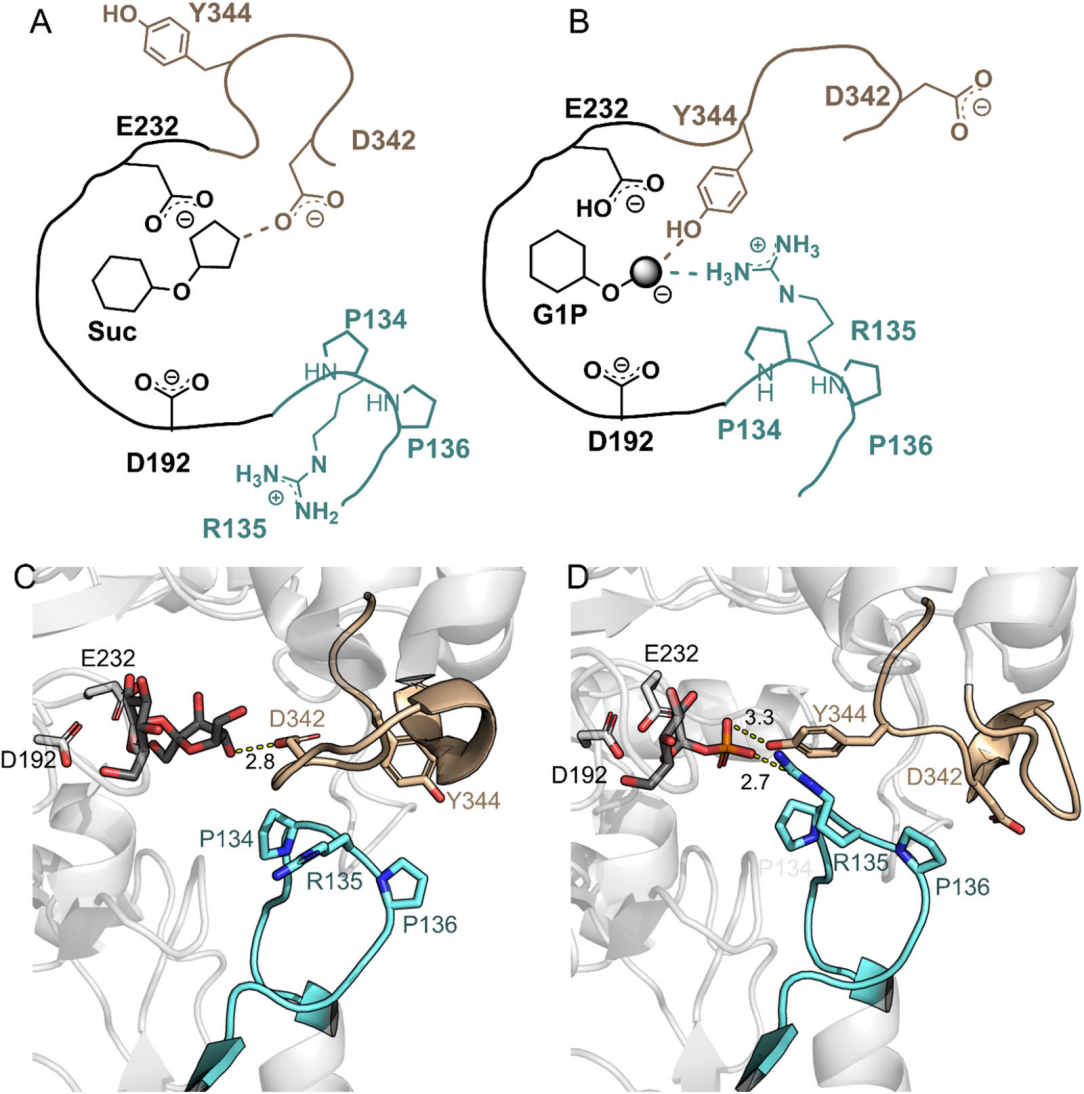
Key elements of protein conformational changes upon binding sucrose (A, C) and α‐d‐glucose 1‐phosphate (B, D) in *Ba*SucP, based on structural data from Mirza et al. (2006). Catalytic residues D192 and E232 function as nucleophile and general acid–base, respectively. Structure in (C) corresponds to PDB ID: 2GDU; (D) shows the G1P binding pose modeled from PDB ID: 2GDV.

The aggregate evidence from Table 1 suggests that P134Q‐*Ba*SucP involves a selective disruption of the enzyme activity for glucosyl transfer to and from phosphate. Enzyme activity for glucosyl transfer from Suc to GOH or water was largely unaffected by the site‐directed substitution. Structural studies of *Ba*SucP (Mirza et al. 2006) revealed a loop rearrangement conformational change used by the enzyme to accommodate fructose and phosphate as leaving group/nucleophile of the reaction. Figure 2 shows schematically the changes in the immediate active site that result from the conformational change. To promote phosphate binding, Arg135 and Tyr344 swing in, and Asp342 swings out of, the binding pocket. In the WT‐*Ba*SucP sequence, Arg135 is flanked by two prolines (Pro134, Pro136) that contribute to a rigid positioning of the arginine in the protein structure. The substitution of Pro134 by Gln is anticipated to interfere with the structural preorganization of the phosphate site specifically. The fructose site might not be affected, providing a structural interpretation consistent with the kinetic data.

Docking analysis (Franceus et al. 2021; Figure S1) showed the probable accommodation of 2GG in the binding pocket of P134Q‐*Ba*SucP. GOH binding by the enzyme variant was suggested to be more specific, and also tighter, than in WT‐*Ba*SucP due to additional hydrogen bonds with the 1‐OH groups of GOH provided. However, the $v_{X}$/$v_{H}$ ratios in Table 1 indicate that WT‐ and P134Q‐*Ba*SucP exhibit similar activities for glycosylation of GOH from Suc. More detailed characterization of P134Q‐*Ba*SucP was therefore required to assess the properties of this enzyme variant relevant for 2GG production.

### 3.2 Initial‐Rate Analysis of Glycosylation of Glycerol

**Figure 3:**
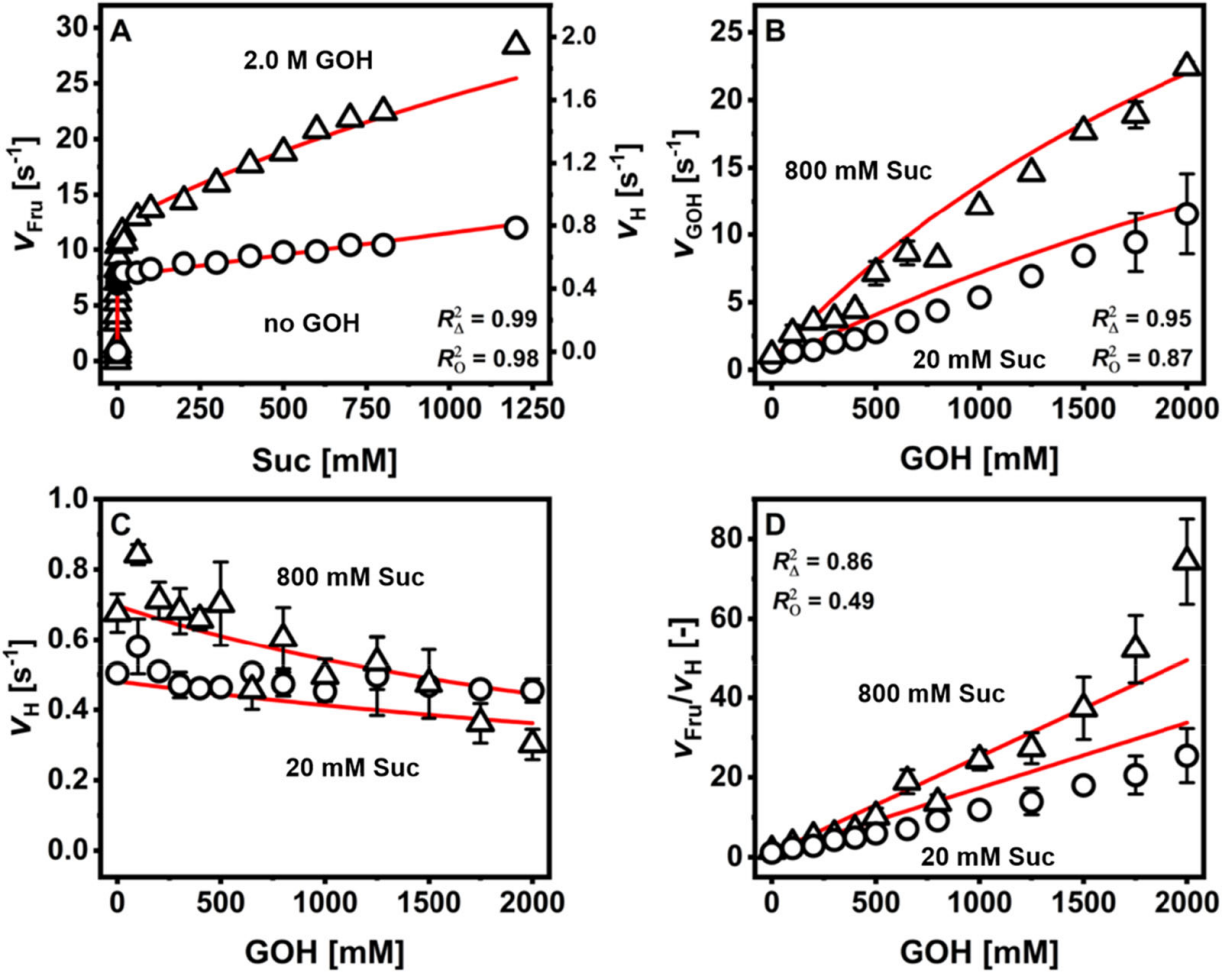
Results of initial‐rate analysis of P134Q‐*Ba*SucP. Symbols show the data, and the lines the fit with the initial rate kinetic model, with goodness of fit indicated. Dependency of $v_{H}$ on [GOH] was not part of the fit but served as internal control. (A) Reactions in the presence of 2.0 M GOH (triangles) and in the absence of GOH (circles). (B–D) Reactions in the presence of 800 (triangles) and 20 mM Suc (circles). Data are averages (*N* = 2), and error bars represent the respective standard deviation.

Initial‐rate study of WT‐*Ba*SucP (Sigg et al. 2021) showed that apparent kinetic parameters for the glycosylation of GOH from Suc (Vfapp, KSucapp, KGOHapp) were dependent on the concentration of the non‐varying donor or acceptor substrate used in the kinetic evaluation. It also showed that apparent kinetic parameters for the hydrolysis (VHapp, KSuc,Happ) were affected when high concentrations of Suc in the range of ~1 M were used. Considering the results for WT‐*Ba*SucP, the kinetic characterization of P134Q‐*Ba*SucP was performed in a similar range of [Suc] (0.05–1200 mM) using no GOH or [GOH] in the range 0–2000 mM. The results are shown in Figure 3, and kinetic parameters obtained from fits of the data with Equations (1)–(4) are summarized in Table 2 along with the corresponding parameters of WT‐*Ba*SucP taken from Sigg et al. (2021).

**Table 2:** Apparent kinetic parameters of WT‐*Ba*SucP and P134Q‐*Ba*SucP.

Kinetic parameter^a^	Suc [mM]	GOH [M]	WT	P134Q
$v_{\text{Fru}}$ [s^−1^]	20	0–2	14.3 ± 1.0	10.6 ± 0.2
$v_{\text{Fru}}$ [s^−1^]	800	0–2	20.9 ± 0.5	22.4 ± 0.4
KGOHapp [M]	20	0–2	≥ 2	≥ 2
	800	0–2	2.50 ± 0.63	≥ 2
KSucapp [mM]	0–20	2	0.45 ± 0.01	1.00 ± 0.07
VSucapp [s^−1^]	0–20	2	15.9 ± 0.1	12.0 ± 0.2
KSucHapp [mM]	0–20	0	0.03 ± 0.006	< 0.1
VSucHapp [s^−1^]	0–20	0	0.87 ± 0.02	0.48 ± 0.01
KGGapp [mM]^b^	0–500	50/500	49.1 ± 6.4	67.1 ± 3.0
VGGapp [s^−1^]^b^	0–500	50/500	3.68·10^−3^ ± 1.25·10^−4^	5.53·10^−3^ ± 1.56·10^−4^
kappcat/KGOHapp [s^−1^M^−1^]	20	0–2	7.05 ± 0.13	5.34 ± 0.13
kappcat/KGOHapp [s^−1^M^−1^]	800	0–2	10.0 ± 0.60	10.6 ± 0.32
$v_{H}$ [s^−1^]	20	0	0.89 ± 0.01	0.50 ± 0.02
	800	0	1.45 ± 0.03	0.68 ± 0.02
	20	2	0.80 ± 0.03	0.45 ± 0.03
	800	2	0.95 ± 0.31	0.30 ± 0.04
TC [M^−1^]^c^	20	0–2	8.0 ± 0.2	11.3 ± 0.2
	800	0–2	12.0 ± 0.6	28.0 ± 1.9

^a^ Determined by fits of Equations (1)–(4) to the experimental data.

^b^ Donor: GG; acceptor: Pi (WT), Fru (P134Q).

^c^ Model calculated 0–2 M.

The dependence of $v_{\text{Fru}}$ on [Suc] at [GOH] of 2.00 M was biphasic (Figure 3A). In the low range of [Suc] ≤ 20 mM, $v_{\text{Fru}}$ increased sharply with increasing [Suc]. Contrary to the expectation of Michaelis–Menten type of enzyme kinetic behavior, $v_{\text{Fru}}$ did not level out at high [Suc] but increased further as the [Suc] was raised. The dependence of $v_{\text{Fru}}$ on [Suc] in the high concentration range was almost linear, with a slope much lower than that of the $v_{\text{Fru}}$ increase at low [Suc] (Figure 3A). The dependence of $v_{\text{Fru}}$ on [Suc] was similarly biphasic for the WT‐*Ba*SucP, as shown in Figure S2A, with the notable difference that for [Suc] ≥ 600 mM the $v_{\text{Fru}}$ decreased again, thus creating a dome‐shaped profile of $v_{\text{Fru}}$ on [Suc].

The dependence of the hydrolysis rate $v_{H}$ on [Suc] (note: no GOH present) was also biphasic, with an overall characteristic that was well comparable between P134Q‐*Ba*SucP (Figure 3A) and WT‐*Ba*SucP (Figure S2A). However, when comparing the two enzymes on the basis of change in $v_{H}$ and $v_{\text{Fru}}$ between low (20 mM) and high (800 mM) [Suc], we realized that $v_{H}$ increased more strongly for WT‐*Ba*SucP, while $v_{\text{Fru}}$ (recorded at 2.00 M of GOH) increased more strongly for P134Q‐*Ba*SucP, in response to the increase in [Suc]. The $v_{\text{Fru}}$/$v_{H}$ ratio would thus be expected to change in favor of glucosyl transfer to glycerol when P134Q‐*Ba*SucP was used (see later). The apparent Michaelis constants (KSucapp, KSucHapp) were higher (~2.2‐fold) for P134Q‐*Ba*SucP, while VSucapp and VSucHapp were slightly lower (1.33‐ and 1.81‐fold, respectively) (Table 2).

We then analyzed the dependence of $v_{\text{Fru}}$ on [GOH] and did so at constant [Suc] of 20 and 800 mM (Figure 3B). Saturation with GOH was not achieved irrespective of the [Suc] used. The result suggests that the KGOHapp exceeded by far the maximum [GOH] used (2.00 M) in the experiments. (Franceus et al. 2021) reported an KGOHapp of 1.85 M for P134Q‐*Ba*SucP, albeit at a higher temperature (52°C instead of 30°C). From the slope of the roughly linear dependence of $v_{\text{Fru}}$ on [GOH], we determined the efficiency of P134Q‐*Ba*SucP for the reaction with GOH (VSucapp/KGOHapp). As shown in Table 2, the kcatapp/KGOHapp increased from low to high [Suc]. P134Q‐*Ba*SucP and WT‐*Ba*SucP were hardly different when compared on the basis of VSucapp/KGOHapp.

We additionally analyzed the dependence of $v_{H}$ on [GOH], revealing a decrease in $v_{H}$ as [GOH] increases (Figure 3C). The effect was stronger at high compared to low [Suc]. Comparing the two enzymes in reactions at [Suc] of 800 mM (Figure 3C, Supporting Information S1: Figure S1B,C), $v_{H}$ was lower (2.13‐fold) and its decrease by [GOH] stronger (1.48‐fold) for P134Q‐*Ba*SucP than WT‐*Ba*SucP. These effects translate directly into rate ratios $v_{\text{Fru}}/v_{H}$ that were higher for P134Q‐*Ba*SucP (Figure 3D) than WT‐*Ba*SucP (Supporting Information S1: Figure S2D). Transfer coefficients (*TC*; Table 2) are the slopes of the dependencies of $v_{\text{Fru}}/v_{H}$ on [GOH] (Figure 3D, Supporting Information S1: Figure S2D). The *TC* value was higher for P134Q‐*Ba*SucP than WT‐*Ba*SucP (Table 2). The effect of the enzyme on *TC* was more pronounced at high (2.33‐fold) compared to low [Suc] (1.41‐fold). These results support the idea that glycosylation of GOH from Suc is enhanced by using P134Q‐*Ba*SucP instead of WT‐*Ba*SucP. However, glycosylation enhancement by the variant enzyme results not from a higher reactivity with GOH (VSucapp/KGOHapp) as one might expect (Franceus et al. 2021), but primarily because suppression of $v_{H}$ at high [GOH] is more efficient in P134Q‐*Ba*SucP (Figure 3C) compared to WT‐*Ba*SucP (Supporting Information S1: Figure S2C).

### 3.3 Time Course Analysis

**Figure 4:**
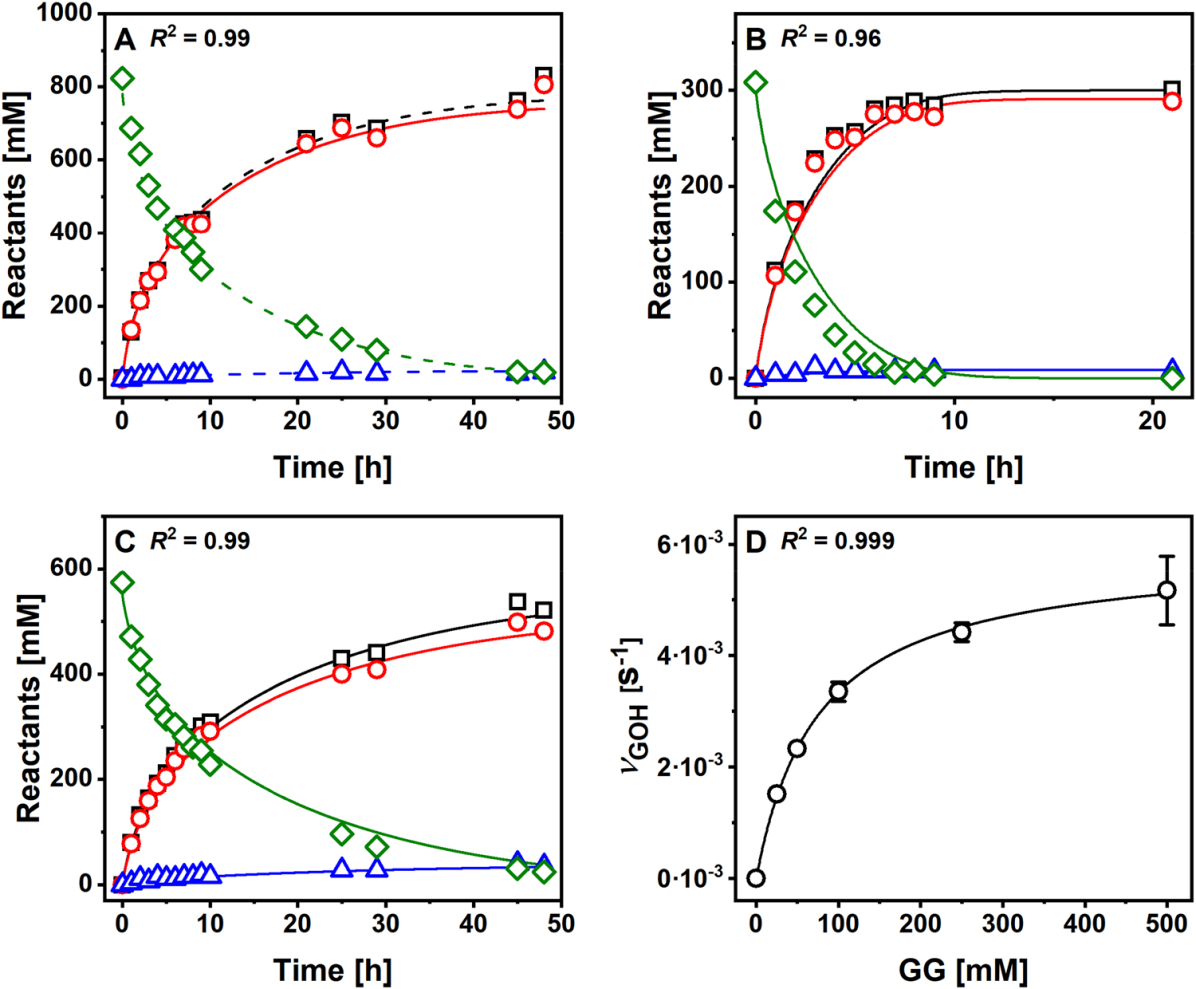
Results of time course analysis for P134Q‐*Ba*SucP. (A–C) [Suc]/[GOH]: 800 mM/2.00 M (A), 300 mM/2.00 M (B), 550 mM/1.00 M (C). Symbols show the data (Suc, diamonds; Fru, squares; GG, circles; Glc, triangles), dashed lines the fit by the hybrid kinetic model, and solid lines corresponding simulations, with goodness of fit (*R* ^2^) indicated. Panel D shows the initial rate of GG phosphorolysis determined at 50 mM Pi. The lines show the fit of Equation 4 with *R* ^2^ indicated. Results of Panel D served as constraints in the fit of data shown in Panel A.

**Figure 5:**
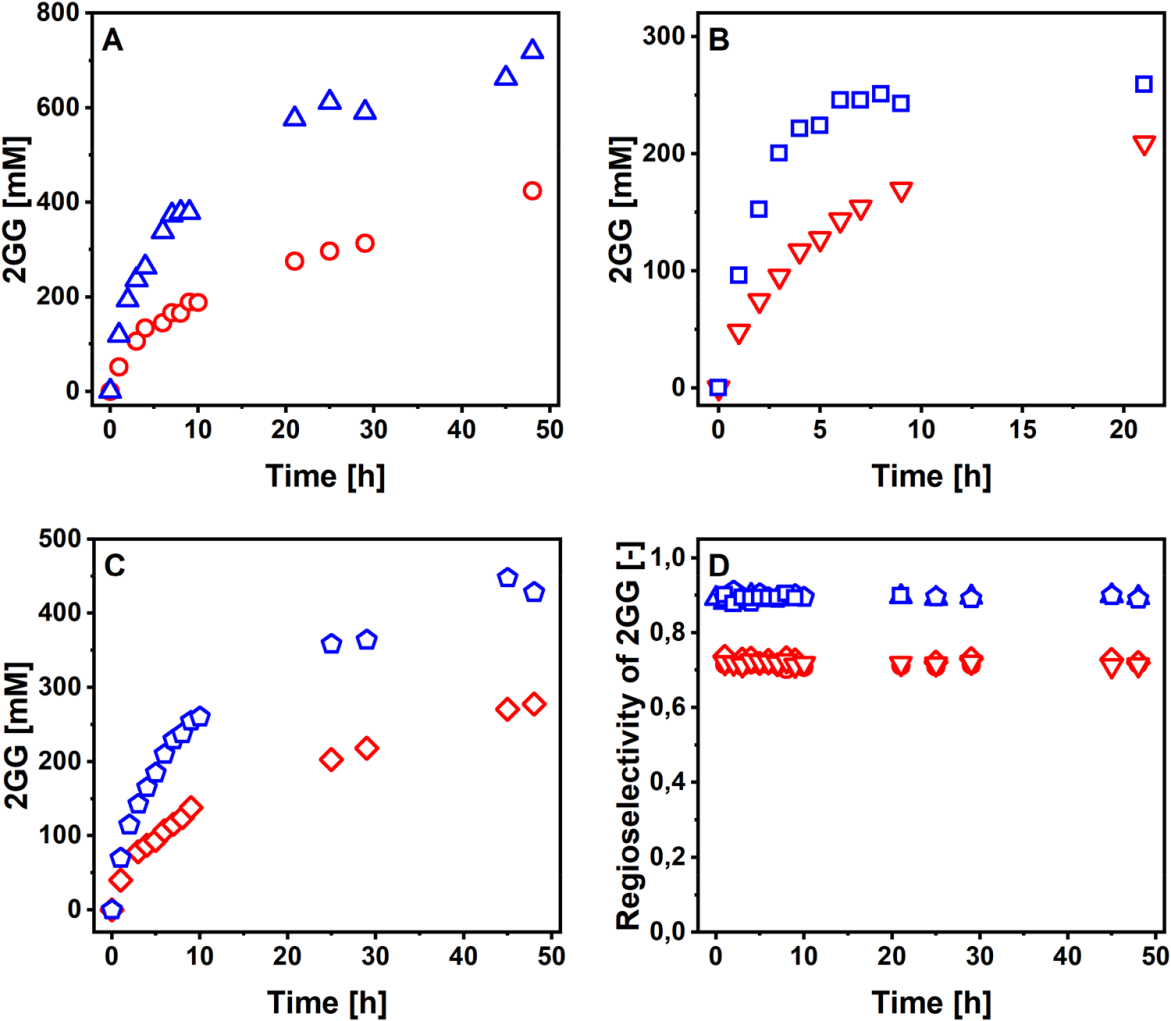
Results for time course analysis of P134Q‐*Ba*SucP‐P134Q (triangles, blue lines) and *Ba*SucP‐WT (circles, red lines). (A–C) Comparison of 2GG production. [Suc]/[GOH]: 800 mM/2.00 M (A), 300 mM/2.00 M (B), 550 mM/1.00 M (C). (D) analysis of $S_{r,2\text{GG}}$ based on data shown in (A–C).

P134Q‐*Ba*SucP was assessed in conversion experiments performed identically as with WT‐*Ba*SucP in Sigg et al. (2021). The substrate concentrations were chosen based on perceived relevance for industrial production, with [GOH] always present in excess over [Suc]. All substrates and products were analyzed by HPLC, and the results are validated on the basis of close mass balance. Reaction time courses are shown in Figure 4 (panels A–C). The corresponding reaction time courses of WT‐*Ba*SucP are found in Figure 6 of Sigg et al. (2021). All reactions of P134Q‐*Ba*SucP reached a sucrose conversion of ≥ 95%, and the release of the hydrolysis product Glc was ≤ 10%. Whereas Figure 4 shows the formation of the total GG released, Figure 5 (panels A–C) shows the formation of the desired 2GG isomer and it does so by comparing reactions of P134Q‐*Ba*SucP and WT‐*Ba*SucP. The large benefit regarding 2GG yield (up to 1.5‐fold) and productivity (up to 1.7‐fold) that results from the use of P134Q‐*Ba*SucP instead of WT‐*Ba*SucP is clear in all reaction conditions. To distinguish between the effect of altered enzyme regioselectivity in the glycosylation of glycerol and general kinetic effects in the enzyme, we analyzed the portion of 2GG in total GG produced, which was found to be 0.89 ± 0.01 and 0.72 ± 0.01 for P134Q‐*Ba*SucP and WT‐*Ba*SucP, respectively (Figure 5D). The results reveal an averaged 2GG/GG ratio (*S*_r_) of 0.89 (±0.01; *N* = 38) and 0.72 (±0.01; *N* = 39) for P134Q‐*Ba*SucP and WT‐*Ba*SucP, respectively. This enzyme‐specific 2GG/GG ratio was invariant with reaction time, hence conversion, as well as with change in the reaction conditions indicated in panels A–C of Figure 5. The enhanced regioselectivity of P134Q‐*Ba*SucP (Franceus et al. 2021) was confirmed by these findings; however, it was shown additionally that the product isomeric purity was not compromised by the progress of the reaction. The relative thermodynamic stabilities of 1GG and 2GG have not been determined to the best of our knowledge. The possibility of enzyme‐catalyzed rearrangement of the kinetically preferred 2GG isomer into 1GG could not be ruled out, thus underlining the importance of the experiments in Figure 5D. The evidence in Figure 5D is relevant furthermore for showing that altered regioselectivity between P134Q‐*Ba*SucP and WT‐*Ba*SucP is not sufficient to account fully for the enhanced 2GG release rate by the variant enzyme. At low sucrose conversion (Figure 5A–C), P134Q‐*Ba*SucP released more than twice the amount of 2GG released by WT‐*Ba*SucP. Comparison of reaction time courses (Figure 4A–C; Figure 6 in Sigg et al. 2021) shows that at an equivalent volumetric loading of enzyme activity, the P134Q‐*Ba*SucP reactions proceeded faster (≥ 1.8‐fold) than the WT‐*Ba*SucP reactions.

**Figure 6:**
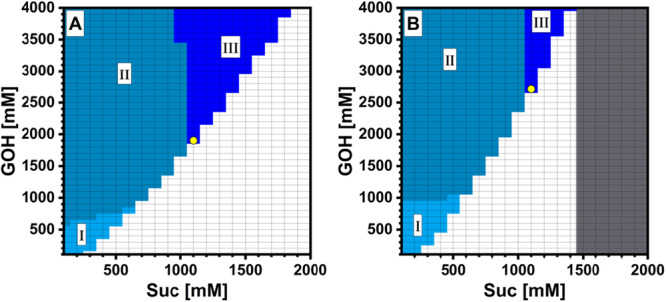
Results of window‐of‐operation analysis for *Ba*SucP‐P134Q (A) and *Ba*SucP‐WT (B) based on simulations generated with the conversion model previously developed. Colored areas refer to reaction conditions at which yield $X_{\text{Suc}}$ ≥ 0.98 (I–III), $Y_{\text{GG}}$ ≥ 0.90 (II–III), and [GG] ≥ 250 g/L (III). The operational optimum is indicated in yellow. The gray area in panel B indicates initial reactions conditions at which $k_{-4}^{*}$ was negative.

In search of an explanation for the superior productivity of the P134Q‐*Ba*SucP reaction, we took from Table 2 that kinetic parameters for the forward reaction from Suc were too similar between the two enzymes to account for the substantially enhanced performance of the P134Q variant. We speculated that the accumulating GG could generate a rate‐retarding effect, and this might not be same for the two enzymes. Initial‐rate kinetic characterization of P134Q‐*Ba*SucP for the reverse reaction from 2GG is shown in Figure 4D, which can be compared to data for WT‐*Ba*SucP in Figure 6D of (Sigg et al. 2021). VGGapp and KGGapp obtained from hyperbolic fit of the data were elevated (~1.4‐fold) in P134Q‐*Ba*SucP compared to WT‐*Ba*SucP. The VGGapp rate was much lower (~10^4^‐fold; Table 2) than the VSucapp rate, suggesting that the reaction from 2GG had little significance. Weaker apparent binding of 2GG reflected in KGGapp suggests that P134Q‐*Ba*SucP could exhibit lower sensitivity to product inhibition than WT‐*Ba*SucP.

### 3.4 Model Fitting

The full hybrid kinetic model of Sigg et al. (2021) was fitted to the data. The fitting procedure was stepwise, beginning with fitting of the initial‐rate model in Supporting Information S1: Table S1 to the data in Figure 3. The results shown in Supporting Information S1: Table S2 were used as parameter start values for, and additionally served to constrain, the fit of the full model to the time courses in Figure 4. Note: the full model differs from the initial‐rate model in that the former includes rate constants for the forward and reverse direction of the reaction, whereas the latter is used for conditions in which the product accumulation is negligible and therefore does not include constants for the reverse reaction. The fitting results are displayed as lines in Figures 4 and 5, and the *R* ^2^ parameter of goodness of fit is shown in the figure panels. Generally, the model provided an excellent fit of the data. Supporting Information S1: Table S3 summarizes the microscopic rate constants of the fitted kinetic model, and Table 3 presents the associated kinetic parameters for P134Q‐*Ba*SucP along with the corresponding parameters for WT‐*Ba*SucP (Sigg et al. 2021). The relationships between kinetic parameters and microscopic rate constants are defined in Supporting Information S1: Table S1. Major differences between P134Q‐*Ba*SucP and WT‐*Ba*SucP are found in the dissociation constants ($K_{\text{iX}}$) and the Michaelis–Menten constants ($K_{X}$) for the substrates and the products. Except for $K_{\text{Fru}}$, the constants were mostly elevated in the enzyme variant, suggesting weakened binding, in the enzyme variant (Table 3). As already mentioned, the increased values of $K_{\text{iGG}}$ (4.0‐fold) and $K_{\text{GG}}$ (1.5‐fold) for P134Q‐*Ba*SucP may be relevant for the variant's higher conversion efficiency compared to WT‐*Ba*SucP. Based on the kinetic parameters in Table 3, the *TC* value was calculated for a [Suc] of 20 and 800 mM, and the results agree reasonably with the approximate values (Table 2) estimated by phenomenological linear fit of the experimental data (Figure 3D). Based on *TC*, P134Q‐*Ba*SucP exhibits superior performance (~1.5‐fold) compared to WT‐*Ba*SucP.

**Table 3:** Kinetic parameters of WT‐*Ba*SucP (Sigg et al. 2021) and P134Q‐*Ba*SucP (this study) calculated from obtained microscopic rate constants (see Supporting Information S1: Tables S2 and S3).

Kinetic parameter	WT	P134Q
$V_{f}$ [s^−1^]	86.4	59.8
$V_{r}$ [s^−1^]^a^	4.78·10^−3^	5.78·10^−3^
$V_{\text{SucH}}$ [s^−1^]	0.97	0.48
$V_{\text{GGH}}$ [s^−1^]^a^	4.76·10^−3^	5.71·10^−3^
$K_{\text{Suc}}$ [mM]	2.58	5.02
$K_{\text{GOH}}$ [mM]	8.91·10^3^	7.58·10^3^
$K_{\text{Fru}}$ [mM]^a^	2.47	0.27
$K_{\text{GG}}$ [mM]^a^	50.8	78.2
$K_{\text{SucH}}$ [mM]	2.90·10^−2^	4.04·10^−2^
$K_{\text{GGH}}$ [mM]^a^	50.5	78.2
$K_{\text{iSuc}}$ [mM]^a^	0.17	11.3
$K_{\text{iGOH}}$ [mM]^a^	100	4.21·10^4^
$K_{\text{iFru}}$ [mM]^a^	14.1	73.3
$K_{\text{iGG}}$ [mM]^a^	50.8	203
$K_{\text{eq}}$ [‐]^a^	8.73·10^3^	700
TC [M^−1^]^b^	10.0/16.0	16.3/24.2

^a^ These kinetic parameters are present only in the reversible model.

^b^ The *TC* is shown for [Suc] of 20 mM/800 mM.

### 3.5 Window‐of‐Operation Analysis for GG Production

The full model (P134Q‐*Ba*SucP: Table 3, Supporting Information S1: Tables S1 and S3; WT‐*Ba*SucP: Sigg et al. (2021)) was used to simulate the enzymatic reactions for constant conditions of activity (11.1 U/mL; determined at [Suc] = 20 mM and [GOH] = 2.00 M) and time (48 h). For the varying conditions, the substrate concentrations were scanned in increments of 100 mM up to 2.00 M for [Suc] and 4.00 M for [GOH]. The processing tasks were defined as follows: conversion $X_{\text{Suc}}$ ≥ 0.98, yield $Y_{\text{GG}}$ ≥ 0.90, and [GG] ≥ 983 mM (250 g/L). The simulation results are displayed as two‐dimensional maps (Figure 6) from which the regions fulfilling one or more specific processing tasks were identified. The operational window of fulfillment of all three processing tasks was ~threefold larger for P134Q‐*Ba*SucP (Figure 6A) than WT‐*Ba*SucP (Figure 6B), which is a clear advantage of the enzyme variant. The optimum operational point of each enzyme was defined from this window by the minimum excess of [GOH] over [Suc] required (see Figure 6). Whereas with both enzymes, the optimum [Suc] was identical at 1.1 M, the demand for GOH differed strongly between P134Q‐*Ba*SucP ([GOH] = 1.9 M) and WT‐*Ba*SucP ([GOH] = 2.7 M). The saving in GOH substrate due to the choice of enzyme was significant (~30%; Δ[GOH] = 0.8 M), and the unreacted GOH at the end of the conversion was decreased by ~twofold to 856 mM when P134Q‐*Ba*SucP was used. We note that separating off the GOH remaining after the reaction represents a major challenge for the 2GG production (Kruschitz and Nidetzky 2020, 2022). The window‐of‐operation analysis shown in Figure 6 does not include the effect of enzyme regioselectivity (*S*_r_). Considering the *S*_r_ value of 0.89 for P134Q‐*Ba*SucP, the 2GG yield ($Y_{2\text{GG}}$) at the optimum operational point was 0.86, which exceeds the $Y_{2\text{GG}}$ of 0.71 for WT‐*Ba*SucP due to the lower *S*_r_, as expected. Interestingly, the $Y_{2\text{GG}}$ for P134Q‐*Ba*SucP also surpassed the corresponding $Y_{2\text{GG}}$ of 0.80 for the naturally specific (*S*_r_ ≥ 0.90), yet less stable SucP from *L. mesenteroides*, as calculated from the literature (Schwaiger et al. 2021; Sigg et al. 2021). Overall, therefore, these considerations reveal the clear application potential of P134Q‐*Ba*SucP as a selective and operationally stable biocatalyst for 2GG production. The example of the industrial production of 2GG in an enzymatic process that employs *Lm*SucP shows the feasibility of successful scale up in principle (Kruschitz and Nidetzky 2022; Luley‐Goedl et al. 2010).

## 4 Conclusions

This study of P134Q‐*Ba*SucP applied to 2GG production makes a general point in showing that enzyme synthetic efficiency in transglycosylation is the result of multiple major factors in a suitable combination (see also Adlercreutz 2017). Detailed assessment of P134Q‐*Ba*SucP in kinetic analyses and conversion studies by experiment and modeling revealed the important interplay of these factors in determining the overall enzyme performance (evaluated in terms of a comprehensive set of metrics of reaction efficiency; Meissner and Woodley 2022). The study shows that besides causing a favorable change in GOH site selectivity that was known from earlier work (Franceus et al. 2021), the site‐directed substitution of Pro134 by Gln additionally causes subtle changes in the kinetic parameters compared to the wild‐type enzyme, which overall provide a significant benefit for the production of 2GG. In particular, the *TC* value was enhanced ~1.5‐fold, which has the important consequence that compared to reaction of the wild‐type enzyme, the excess GOH used in the process can be decreased. Protein engineering approaches that specifically target the *TC* for transglycosylation catalyst optimization (e.g., Lundemo et al. 2017; Teze et al. 2021) are thus supported in general. Recommendation from the study of P134Q‐*Ba*SucP, however, is that the *TC* should be evaluated under the same assay conditions, or at least reasonably similar to, the conditions used in the process. The reason is that due to particular effects of the donor and acceptor concentrations on the rates of transfer and hydrolysis, the *TC* may show substantial variation with the reaction conditions used. In summary, P134Q‐*Ba*SucP combines favorable properties of selectivity, reactivity, and stability for possible application in the glycosylation of GOH from Suc to further improve biocatalytic production of 2GG (Kruschitz and Nidetzky 2022; Luley‐Goedl et al. 2010). Mechanistically, it was interesting to find that P134Q‐*Ba*SucP involves disruption of the reactivity with phosphate. The important use of kinetic modeling to optimize enzymatic transglycosylations is shown (see also, Fagundes et al. 2022; Mitchell and Krieger 2022; Weber et al. 2024). SucP exhibits broad specificity for the acceptor substrate used in the transglycosylation from Suc (Franceus and Desmet 2020). SucPs from different sources were also engineered for improved performance (e.g., activity, selectivity) in the reaction with different acceptors (for review, see Franceus and Desmet 2020; Zhu et al. 2025). The analysis presented here can be relevant for the characterization and the optimization of the various synthetic reactions catalyzed by wild‐type and engineered forms of SucP (see also Klimacek et al. 2020; Sigg et al. 2021).

## Author Contributions

**Alexander Sigg, Mario Klimacek,** and **Bernd Nidetzky:** study design. **Alexander Sigg:** experiments. **Alexander Sigg** and **Mario Klimacek:** data analysis, modeling. **Martin Pfeiffer** and **Jorick Franceus:** protein modeling, visualization. **Jorick Franceus** and **Tom Desmet:** resources. **Alexander Sigg** and **Bernd Nidetzky:** writing, discussion, editing. **Tom Desmet** and **Bernd Nidetzky:** supervision, funding acquisition.

## Conflicts of Interest

The authors declare no conflicts of interest.

## Supporting information

Revision P134Q SI 10Apr.

## Data Availability

The data that support the findings of this study are available from the corresponding author upon reasonable request.
