# Supplementary material for: The P134Q Sucrose Phosphorylase: Subtle Changes in the Catalytic Properties Benefit the Production of 2‐O‐α‐Glucosyl Glycerol
Source: Biotechnol Bioeng. 2025 Jun 20;122(9):2465–77. doi: 10.1002/bit.70003 (PMC12322648; doi:10.1002/bit.70003)
Supplement: Supplementary file 1 — Revision P134Q SI 10Apr. [file BIT-122-2465-s001.docx]

**SUPPORTING INFORMATION**

**The P134Q sucrose phosphorylase: subtle changes in the catalytic properties benefit the production of 2-*O*-α-glucosyl glycerol**

Alexander SIGG^1,2^, Mario KLIMACEK^1^, Martin PFEIFFER^1,2^, Jorick FRANCEUS^3^, Tom DESMET^2,3^, and Bernd NIDETZKY^1,2,*^

^1^ Institute of Biotechnology and Biochemical Engineering, Graz University of Technology, NAWI Graz, Petersgasse 10 – 12, A-8010 Graz, Austria

^2^ Austrian Centre of Industrial Biotechnology (acib), Petersgasse 14, A-8010 Graz, Austria

^3^ Centre for Synthetic Biology (CSB), Unit for Biocatalysis and Enzyme Engineering, Faculty of Bioscience Engineering, Ghent University, Coupure Links 653, 9000 Gent, Belgium

* Corresponding author (B.N.), e-mail: bernd.nidetzky@tugraz.at; phone: +433168738400

| Table S1. Description of the hybrid model used for the fit of initial rate and time course data taken from (Sigg et al., 2021) Microscopic rate constants are numbered according to the kinetic mechanism shown in Figure 1B. $k_{-2}$ and $k_{-4}$ were not part of the initial rate model | | | | | | | | | | |
| --- | --- | --- | --- | --- | --- | --- | --- | --- | --- | --- |
| **Microscopic rate constants** | | | | | | | | | | |
|  | $k_{+1}\left[ \mathrm{Suc} \right]$  $k_{-1}$  $k_{+2}$  $k_{-2}[Fru]$  $k_{+3}^{*}[GOH]$  $k_{-3}$  $k_{+4}^{*}$  $k_{-4}[\mathrm{GG}]$  $k_{+5}^{*}$ | | | | mM^-1^s^-1^  s^-1^  s^-1^  mM^-1^s^-1^  mM^-1^s^-1^  s^-1^  s^-1^  mM^-1^s^-1^  s^-1^ | | | E + Suc → E·Suc  E·Suc → E + Suc  E·Suc → E-Glc + Fru  E-Glc + Fru → E·Suc  E-Glc + GOH → E·GG  E·GG → E-Glc + GOH  E·GG → E + GG  E + GG → E + GG  E-Glc → E + Glc | | |
|  | with | | | | | | | | |  |
|  | $k_{i}^{*}$ | { | $\left[ \mathrm{Suc} \right]\leq20mM;k_{i}^{*}=k_{i}$  $\left[ \mathrm{Suc} \right]>20mM;k_{i}^{*}=k_{i}+a_{i}(\left[ \mathrm{Suc} \right]-20mM])$ | | | | | | | |
|  |  |  |  |  |  |  |  |  |  |  |
|  | | | | | | | | | | |
| **Rate equations based on kinetic parameters** | | | | | | | | | | |
| $v_{\mathrm{Fru}}=\frac{d\left[ \mathrm{Fru} \right]}{\mathrm{dt}}=\frac{V_{r}V_{f}\left( \left[ \mathrm{Suc} \right]\left[ \mathrm{GOH} \right]-\frac{\left[ \mathrm{Fru} \right]\left[ \mathrm{GG} \right]}{K_{\mathrm{eq}}} \right)+V_{r}V_{\mathrm{SucH}}K_{\mathrm{GOH}}\left[ \mathrm{Suc} \right]}{D}$ | | | | | | | $v_{\mathrm{GG}}=\frac{d\left[ \mathrm{GG} \right]}{\mathrm{dt}}=\frac{V_{r}V_{f}\left( \left[ \mathrm{Suc} \right]\left[ \mathrm{GOH} \right]-\frac{\left[ \mathrm{Fru} \right]\left[ \mathrm{GG} \right]}{K_{\mathrm{eq}}} \right)-\frac{V_{f}V_{\mathrm{GGH}}K_{\mathrm{Fru}}\left[ \mathrm{GG} \right]}{K_{\mathrm{eq}}}}{D}$ | | | |
| $v_{\mathrm{Suc}}=\frac{d\left[ \mathrm{Suc} \right]}{\mathrm{dt}}=\frac{V_{r}V_{f}\left( -\left[ \mathrm{Suc} \right]\left[ \mathrm{GOH} \right]+\frac{\left[ \mathrm{Fru} \right]\left[ \mathrm{GG} \right]}{K_{\mathrm{eq}}} \right)-V_{r}V_{\mathrm{SucH}}K_{\mathrm{GOH}}\left[ \mathrm{Suc} \right]}{D}$ | | | | | | | $v_{\mathrm{GOH}}=\frac{d\left[ \mathrm{GOH} \right]}{\mathrm{dt}}=\frac{V_{r}V_{f}\left( -\left[ \mathrm{Suc} \right]\left[ \mathrm{GOH} \right]+\frac{\left[ \mathrm{Fru} \right]\left[ \mathrm{GG} \right]}{K_{\mathrm{eq}}} \right)+\frac{V_{f}V_{\mathrm{GGH}}K_{\mathrm{Fru}}\left[ \mathrm{GG} \right]}{K_{\mathrm{eq}}}}{D}$ | | | |
| $v_{H}=\frac{d[Glc]}{\mathrm{dt}}=\frac{V_{r}V_{\mathrm{SucH}}K_{\mathrm{GOH}}\left[ \mathrm{Suc} \right]+\frac{V_{f}V_{\mathrm{GGH}}K_{\mathrm{Fru}}\left[ \mathrm{GG} \right]}{K_{\mathrm{eq}}}}{D}$ | | | | | | | | | | |
| with | | | | | |  | | | | |
| $D=V_{r}K_{\mathrm{GOH}}\left[ \mathrm{Suc} \right]+V_{r}K_{\mathrm{Suc}}\left[ \mathrm{GOH} \right]+\frac{K_{\mathrm{GG}}V_{f}}{K_{\mathrm{eq}}}\left[ \mathrm{Fru} \right]+\frac{K_{\mathrm{Fru}}V_{f}}{K_{\mathrm{eq}}}\left[ \mathrm{GG} \right]+V_{r}\left[ \mathrm{Suc} \right]\left[ \mathrm{GOH} \right]+\frac{K_{\mathrm{GG}}V_{f}}{K_{\mathrm{iSuc}}K_{\mathrm{eq}}}\left[ \mathrm{Suc} \right]\left[ \mathrm{Fru} \right]+\frac{V_{f}}{K_{\mathrm{eq}}}\left[ \mathrm{Fru} \right]\left[ \mathrm{GG} \right]$   $+\frac{K_{\mathrm{Suc}}V_{r}}{K_{\mathrm{iGG}}}\left[ \mathrm{GOH} \right]\left[ \mathrm{GG} \right]+K_{\mathrm{SucH}}K_{\mathrm{GOH}}V_{r}$ a | | | | | | | | | | |
|  | | | | | | | | | | |
| **Kinetic parameter expressed with microscopic rate constants** | | | | | | | | | | |
| Michaelis constants | | | | Maximum velocity | | | | | Dissociation constants: | |
| $K_{\mathrm{Suc}}=\frac{k_{+4}^{*}\left( k_{-1}+k_{+2} \right)}{k_{+1}\left( k_{+2}+k_{+4}^{*} \right)}$ | | | | $V_{f}=\frac{k_{+2}k_{+4}^{*}}{k_{+2}+k_{+4}^{*}}$ | | | | | $K_{\mathrm{iSuc}}=\frac{k_{-1}}{k_{+1}}$ | |
| $K_{\mathrm{GOH}}=\frac{\left( k_{+2}+k_{+5}^{*} \right)\left( k_{-3}+k_{+4}^{*} \right)}{k_{+3}^{*}\left( k_{+2}+k_{+4}^{*} \right)}$ | | | | $V_{r}=\frac{k_{-1}\cdot k_{-3}}{k_{-1}+k_{-3}}$ | | | | | $K_{\mathrm{iGOH}}=\frac{k_{-3}+k_{+5}^{*}}{k_{+3}^{*}}$ | |
| $K_{\mathrm{Fru}}=\frac{\left( k_{-3}+k_{+5}^{*} \right)\left( k_{-1}+k_{+2} \right)}{k_{-2}\left( k_{-1}+k_{-3} \right)}$ | | | | $V_{\mathrm{SucH}}=\frac{k_{+2}k_{+5}^{*}}{k_{+2}+k_{+5}^{*}}$ | | | | | $K_{\mathrm{iFru}}=\frac{k_{+2}+k_{+5}^{*}}{k_{-2}}$ | |
| $K_{\mathrm{GG}}=\frac{k_{-1}\left( k_{-3}+k_{+4}^{*} \right)}{k_{-4}\left( k_{-1}+k_{-3} \right)}$ | | | | $V_{\mathrm{GGH}}=\frac{k_{-3}k_{+5}^{*}}{k_{-3}+k_{+5}^{*}}$ | | | | | $K_{\mathrm{iGG}}=\frac{k_{+4}^{*}}{k_{-4}}$ | |
| $K_{\mathrm{SucH}}=\frac{k_{+5}^{*}\left( {k_{-1}+k}_{+2} \right)}{k_{+1}\left( k_{+2}+k_{+5}^{*} \right)}$ | | | | Equilibrium constant and Haldane relationships | | | | | | |
| $K_{\mathrm{GGH}}=\frac{k_{+5}^{*}\left( {k_{-3}+k}_{+4}^{*} \right)}{k_{-4}\left( k_{-3}+k_{+5}^{*} \right)}$ | | | | $K_{\mathrm{eq}}=\frac{k_{+1}k_{+2}k_{+3}^{*}k_{+4}^{*}}{k_{-1}k_{-2}k_{-3}k_{-4}}=\frac{V_{f}K_{\mathrm{GG}}K_{\mathrm{iFru}}}{V_{r}K_{\mathrm{GOH}}K_{\mathrm{iSuc}}}=\frac{V_{f}{K_{\mathrm{Fru}}K}_{\mathrm{iGG}}}{V_{r}K_{\mathrm{Suc}}K_{\mathrm{iGOH}}}=\frac{V_{f}K_{\mathrm{Fru}}K_{\mathrm{GGH}}}{V_{r}K_{\mathrm{GOH}}K_{\mathrm{SucH}}}$ | | | | | | |
|  | | | |  | | | | |  | |
| **Rate ratios** | | | | | | | | | | |
| $\frac{v_{\mathrm{GG}}}{v_{H}}=\frac{V_{r}V_{f}\left( \left[ \mathrm{Suc} \right]\left[ \mathrm{GOH} \right]-\frac{\left[ \mathrm{Fru} \right][\mathrm{GG}]}{K_{\mathrm{eq}}} \right)-\frac{V_{f}V_{\mathrm{rH}}K_{\mathrm{Fru}}}{K_{\mathrm{eq}}}[\mathrm{GG}]}{V_{r}V_{\mathrm{fH}}K_{\mathrm{GOH}}\left[ \mathrm{Suc} \right]+\frac{V_{f}V_{\mathrm{rH}}K_{\mathrm{Fru}}}{K_{\mathrm{eq}}}[\mathrm{GG}]}=\frac{k_{+3}k_{+2}k_{+4}\left[ \mathrm{Suc} \right]\left[ \mathrm{GOH} \right]-\frac{k_{-3}k_{-4}}{k_{+1}}k_{-2}k_{-1}\left[ \mathrm{Fru} \right]\left[ \mathrm{GG} \right]-\frac{k_{-3}k_{-4}}{k_{+1}}k_{+5}\left( k_{-1}+k_{+2} \right)[\mathrm{GG}]}{k_{+5}\left( k_{+2}\left( k_{-3}+k_{+4} \right)\left[ \mathrm{Suc} \right]+\frac{k_{-3}k_{-4}}{k_{+1}} \left( k_{-1}+k_{+2} \right)\left[ \mathrm{GG} \right] \right)}$ | | | | | | | | | | |
| $\frac{v_{\mathrm{Fru}}}{v_{H}}=\frac{V_{r}V_{f}\left( \left[ \mathrm{Suc} \right]\left[ \mathrm{GOH} \right]-\frac{\left[ \mathrm{Fru} \right][\mathrm{GG}]}{K_{\mathrm{eq}}} \right)-V_{r}V_{\mathrm{fH}}K_{\mathrm{GOH}}[\mathrm{Suc}]}{V_{r}V_{\mathrm{fH}}K_{\mathrm{GOH}}\left[ \mathrm{Suc} \right]+\frac{V_{f}V_{\mathrm{rH}}K_{\mathrm{Fru}}}{K_{\mathrm{eq}}}[\mathrm{GG}]}=\frac{k_{+3}k_{+2}k_{+4}\left[ \mathrm{Suc} \right]\left[ \mathrm{GOH} \right]-\frac{k_{-3}k_{-4}}{k_{+1}}k_{-2}k_{-1}\left[ \mathrm{Fru} \right]\left[ \mathrm{GG} \right]-k_{+2}k_{+5}\left( k_{-3}+k_{+4} \right)[\mathrm{Suc}]}{k_{+5}\left( k_{+2}\left( k_{-3}+k_{+4} \right)\left[ \mathrm{Suc} \right]+\frac{k_{-3}k_{-4}}{k_{+1}} \left( k_{-1}+k_{+2} \right)\left[ \mathrm{GG} \right] \right)}$ | | | | | | | | | | |
| Abbreviations used: Suc, sucrose; Fru fructose; GOH, glycerol; GG, α- glucosyl glycerol; Glc, glucose; $V_{f}$ and $V_{r}$, maximal velocity in the forward and reverse direction, index H is for hydrolysis ( $k_{+5}^{*}$); $K$, Michaelis constants; $K_{i}$, dissociation constant; $K_{\mathrm{eq}}$, equilibrium constant $v$, reaction rate of respective component; $k$, microscopic rate constant; $a$, linear rate constant modifier; ${}^{*}$, modified rate constant. | | | | | | | | | | |

| Table S2. Fitting results of initial rate data applying the initial rate model as stated in Table S1 for *Ba*SucP-P134Q. The best fit result is shown with (minimum / maximum) values obtained in *N*=10 fitting runs. | | | | | |
| --- | --- | --- | --- | --- | --- |
|  | |  | Boundaries | | Estimates |
| Microscopic rate constant | | | lb | ub |  |
| $k_{+1}\left[ \mathrm{Suc} \right]$ | [mM^-1^s^-1^] | | 1.00 | 10^20^ | **75.3** (11.0 / 75.3) |
| $k_{-1}$ | [s^-1^] | | 10^-20^ | 10^20^ | **737** (10^-20^ / 1.25·10^3^) |
| $k_{+2}$ | [s^-1^] | | 10^-20^ | 10^3 b^ | **128** (128 / 10^3^) |
| $k_{+3}\left[ \mathrm{GOH} \right]$ | [mM^-1^s^-1^] | | 10^-10^ | 10^20^ | **5.63·10^-3^** (5.43·10^-3^ / 5.66·10^-3^) |
| $a_{+3}$ | [mM^-2^s^-1^] | | 10^-20^ | 10^20^ | **1.01·10^-5^** (8.08·10^-6^ / 1.13·10^-5^) |
| $k_{-3}$ | [s^-1^] | | 10^-20^ | 10^-2^ | **1.00·10^-2^** (1.00·10^-20^ / 1.00·10^-2^) |
| $k_{+4}$ | [s^-1^] | | 10^-20^ | 10^20^ | **2.56·10^6^** (157 / 2.56·10^6^) |
| $a_{+4}$ | [mM^-1^s^-1^] | | -10^20^ | -10^-20^ | -**3.20·10^4^** (-3.20·10^4^ / 10^-20^) |
| $k_{+5}$ | [s^-1^] | | 10^-10^ | 3.00 | **0.52** (0.51 / 0.52) |
| $a_{+5}$ | [mM^-1^s^-1^] | | 10^-20^ | 10^20^ | **1.29·10^-4^** (5.67·10^-5^ / 1.75·10^-4^) |
|  |  | |  |  |  |
| lb and ub: lower and upper boundaries. | | | | | |

| Table S3. Results from fitting time course data with the hybrid model for *Ba*SucP-P134Q. The best fit result is shown with (minimum / maximum) values obtained in *N*=10 fitting runs. Microscopic rate constants and initial reaction conditions were fitted, while results from initial rate analysis served as additional model restrictions. Estimates from initial rate parameter estimation served as start values (see Table S2). | | | | | | |
| --- | --- | --- | --- | --- | --- | --- |
|  | |  | Boundaries | | | Estimates |
| Microscopic rate constant | | | lb | | ub |  |
| $k_{+1}\left[ \mathrm{Suc} \right]$ | [mM^-1^s^-1^] | | 10^-6^ | | 10^6^ | **99.8** (46.0 / 5.66·10^3^) |
| $k_{-1}$ | [s^-1^] | | 10^-6^ | | 10^6^ | **1128** (461 / 4.87·10^4^) ^a^ |
| $k_{+2}$ | [s^-1^] | | 10^-6^ | | 10^3 b^ | **153** (72.5 / 161) |
| $k_{-2}\left[ \mathrm{Fru} \right]$ | [mM^-1^s^-1^] | | 10^-6^ | | 10^6^ | **2.09** (1.15 / 2.25) |
| $k_{+3}\left[ \mathrm{GOH} \right]$ | [mM^-1^s^-1^] | | 10^-4^ | | 1.00 | **7.92·10^-3^** (7.22·10^-3^ / 8.01·10^-3^) |
| $a_{+3}$ | [mM^-2^s^-1^] | | 10^-7^ | | 1.00 | **1.16·10^-5^** (1.03·10^-5^ / 1.1.29·10^-5^) |
| $k_{-3}$ | [s^-1^] | | 10^-20^ | | 0.01 | **5.78·10^-3^** (5.78·10^-3^ / 5.98·10^-3^) |
| $k_{+4}$ | [s^-1^] | | 10^-6^ | | 10^6^ | **98.3** (89.1 / 2.67·10^4^) |
| $a_{+4}$ | [mM^-1^s^-1^] | | -10^20^ | | -10^-20^ | -**1.24·10^-8^** (-3.14 / -2.91·10^-2^) |
| $k_{-4}\left[ \mathrm{GG} \right]$ | [mM^-1^s^-1^] | | 10^-6^ | | 1000 | **1.24** (1.24 / 370) |
| $k_{+5}$ | [s^-1^] | | 10^-6^ | | 1 | **0.48** (0.48 / 0.49) |
| $a_{+5}$ | [mM^-1^s^-1^] | | 10^-6^ | | 2‧10^-3^ | **2.79·10^-4^** (2.27·10^-4^ / 2.81·10^-4^) |
|  |  | |  | |  |  |
| Initial reaction condition | |  |  | |  |  |
| $E_{0}$ | [μM] | | 2.56 | | 3.13 | **3.13** |
| $\left[ \mathrm{Suc} \right]_{0}$ | [mM] | | 780 | | 820 | **780** |
| $\left[ \mathrm{GOH} \right]_{0}$ | [M] | | 1.95 | | 2.05 | **2050** |
|  |  | |  | |  |  |
| Model restrictions applied to PE | | | |  |  |  |
| ${}^{\mathrm{app}}{K_{\mathrm{Suc}}}$ | [mM] | | 0.94 ^a^ | | 1.08 ^a^ | **1.08** |
| ${}^{\mathrm{app}}{K_{\mathrm{GOH}}}$ | [M] | | 2.00 ^b^ | | 10^20^ ^b^ | **6.07** |
| ${}^{\mathrm{app}}{K_{\mathrm{GG}}}$ | [mM] | | 72.1 ^c^ | | 79.1 ^c^ | **79.1** |
| ${}^{\mathrm{app}}{V_{\mathrm{GG}}}$ | [s^-1^] | | 5.78·10^-3 c^ | | 5.95·10^-3 c^ | **5.78·10^-3^** |
| $v_{\mathrm{Fru}}^{\left[ \mathrm{Suc} \right]=20mM}$ | [s^-1^] | | 11.8 ^d^ | | 12.2 ^d^ | **12.2** |
| $v_{\mathrm{Fru}}^{\left[ \mathrm{Suc} \right]=800mM}$ | [s^-1^] | | 22.0 ^d^ | | 22.9 ^d^ | **22.0** |
| $v_{\mathrm{SucH}}^{\left[ \mathrm{Suc} \right]=20mM}$ | [s^-1^] | | 0.48 ^e^ | | 0.52 ^e^ | **0.48** |
| $v_{\mathrm{SucH}}^{\left[ \mathrm{Suc} \right]=800mM}$ | [s^-1^] | | 0.67 ^e^ | | 0.70 ^e^ | **0.70** |
| lb and ub: lower and upper boundaries.  ^a^ [Suc] ≤ 20 mM, $[$GOH] = 2000 mM  ^b^ [Suc] = 20 mM, $[$GOH] ≤ 2000 mM  ^c^ Results shown in Figure 4D served as model restriction  ^d^ [GOH] = 2000 mM  ^e^ [GOH] = 0 | | | | | | |


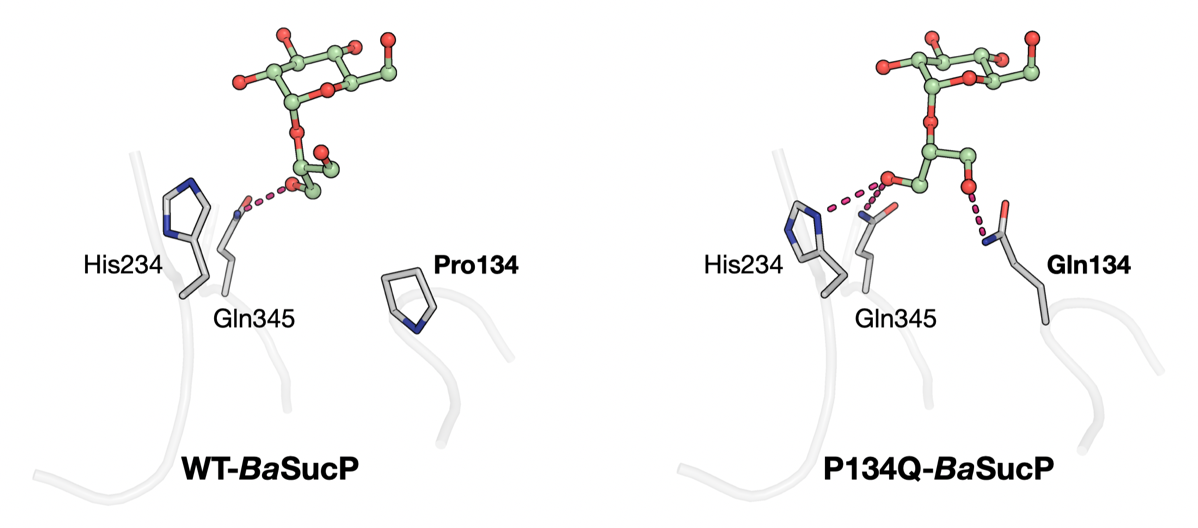


**Figure S1.** Docking of 2-GG in the active site of BaSucP (PDB ID: 2GDV, molecule B) and in a model of variant P134Q-BaSucP. The results are from Franceus et al. (2021).


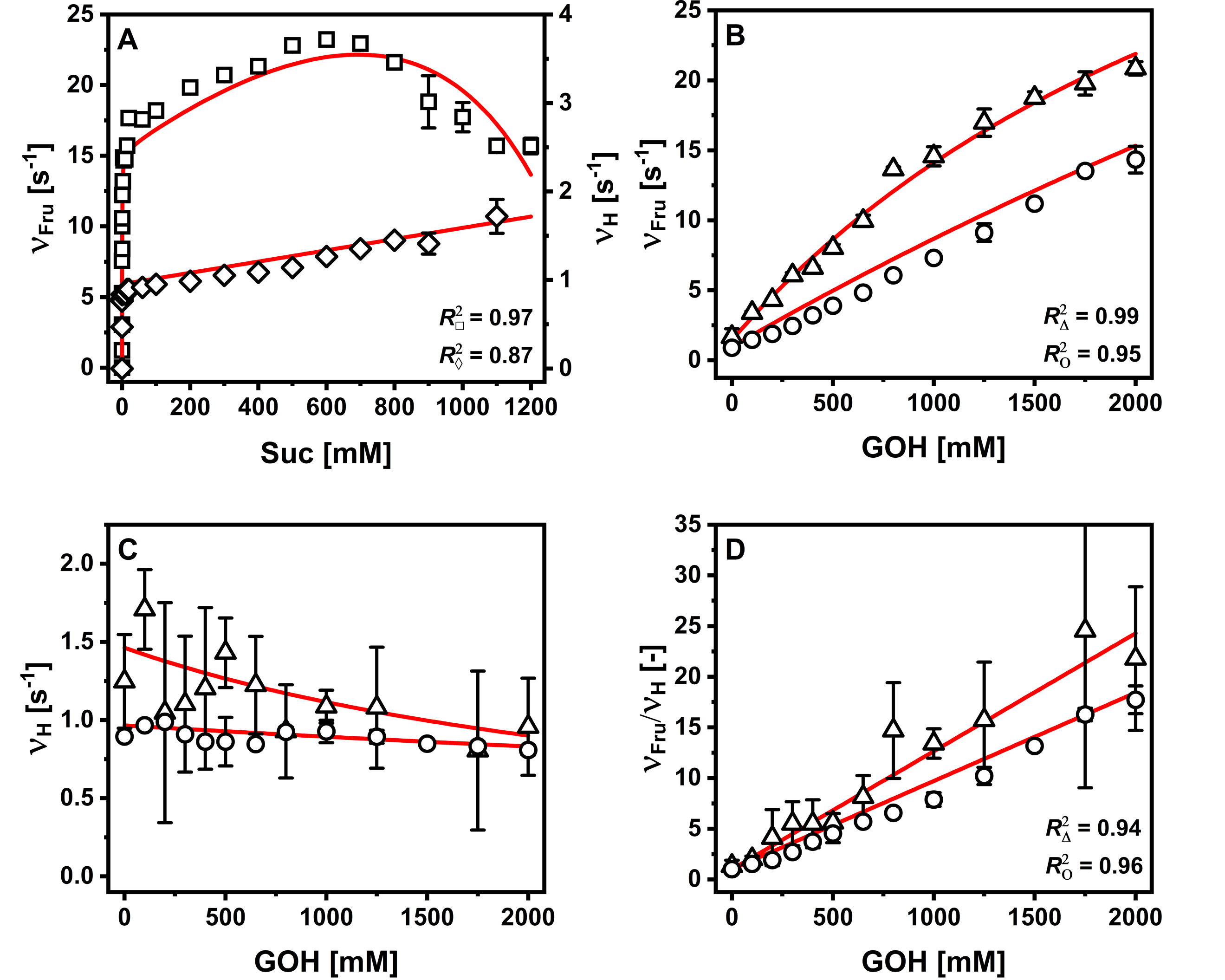


**Figure S2.** Initial rate data for the wild-type of *Ba*SucP taken from Klimacek et al. (2020). Symbols show the data (Panel A: squares, [GOH] = 2.00 M; diamonds, [GOH] = 0; Panel B – D: triangles, [Suc] = 800 mM; circles, [Suc] = 20 mM). Data are averages (*N* = 2) and error bars represent the corresponding standard deviation. Lines show fit of the respective initial rate model with goodness of fit indicated (*R*^2^). The dependency of $v_{H}$ on [GOH] was not fitted and shows results of simulations.

**References**

Franceus, J., Ubiparip, Z., Beerens, K., & Desmet, T. (2021). Engineering of a thermostable biocatalyst for the synthesis of 2-O-glucosylglycerol. *Chembiochem*, *22*(18), 2777-2782. <https://doi.org/10.1002/cbic.202100192>

Klimacek, M., Sigg, A., & Nidetzky, B. (2020). On the donor substrate dependence of group-transfer reactions by hydrolytic enzymes: Insight from kinetic analysis of sucrose phosphorylase-catalyzed transglycosylation. *Biotechnol Bioeng*, *117*(10), 2933-2943. <https://doi.org/10.1002/bit.27471>

Sigg, A., Klimacek, M., & Nidetzky, B. (2021). Three-level hybrid modeling for systematic optimization of biocatalytic synthesis: alpha-glucosyl glycerol production by enzymatic trans-glycosylation from sucrose. *Biotechnol Bioeng*, *118*(10), 4028-4040. <https://doi.org/10.1002/bit.27878>
